# Supplementary material for: Spatial Distribution, Source Analysis and Health Risk Study of Heavy Metals in the Liujiang River Basin in Different Seasons
Source: Int J Environ Res Public Health. 2022 Nov 22;19(23):15435. doi: 10.3390/ijerph192315435 (PMC9738270; doi:10.3390/ijerph192315435)
Supplement: Supplementary file 1 [file ijerph-19-15435-s001.zip › ijerph-2019628-supplementary.pdf]

Table S1. Distributions of parameters for the health risk assessment of heavy metals [31,35,69,70].

	adults	children
IR	2.2 L/day	1 L/day
ED	70 years	6 years
Bw	65.3 kg	21.3 kg
SA	18,000 cm^2	6600 cm^2
ET	0.58 h/day	1 h/day
EF	365 days/year	
CF	1×10^{-3}	

Table S2. Reference dose (RfD) and dermal permeability coefficient in samples (Kp), and slope factor (SF) of heavy metal(loid)s used for health risk assessment. [30,31,37,54,71]

	$RfD_{ingestion}$(ug/kg/day)	RfD_{dermal}(ug/kg/day)	Kp (cm/h)	$SF_{ingstion}$ $(mg/kg/d)^{-1}$	SF_{dermal} $(mg/kg/d)^{-1}$
Mn	140	0.96	1×10^{-3}	–	–
Ni	20	0.8	2×10^{-4}	1.7	42.5
Pb	1.4	0.42	1×10^{-4}	8.5×10^{-3}	0.073
Zn	300	60	6×10^{-4}	–	–
As	0.3	0.123	1×10^{-3}	1.5	1.5
Cr	3	0.075	1×10^{-3}	0.5	20
Cd	0.5	0.025	1×10^{-3}	6.1	0.38
Cu	5	8	1×10^{-3}	–	–
Co	300	0.06	4×10^{-4}	–	–
Hg	0.3	0.021	1×10^{-3}	–	–
Al	0.4	200	1×10^{-3}	–	–

Table S3. Reference dose, Hazard quotient and cancer risk for each element of the Liujiang River, China.

Element	$HI_{ingenstion}$		HI_{dermal}		THI		Cancer $risk_{ingenstion}$		Cancer $risk_{dermal}$		TCR	
	Adult	Child	Adult	Child	Adult	Child	Adult	Child	Adult	Child	Adult	Child
（a）Dry season in R1												
As	1.477×10^{-1}	2.058×10^{-1}	1.709×10^{-3}	3.312×10^{-3}	1.494×10^{-1}	2.091×10^{-1}	6.645×10^{-5}	9.260×10^{-5}	3.153×10^{-7}	6.111×10^{-7}	6.676×10^{-5}	9.321×10^{-5}
Cd	9.613×10^{-3}	1.340×10^{-2}	9.124×10^{-4}	1.768×10^{-3}	1.053×10^{-2}	1.517×10^{-2}	2.932×10^{-5}	4.086×10^{-5}	8.668×10^{-9}	1.680×10^{-8}	2.933×10^{-5}	4.088×10^{-5}
Zn	1.602×10^{-3}	5.342×10^{-3}	1.637×10^{-5}	3.173×10^{-5}	1.619×10^{-3}	5.373×10^{-3}						

Cr	1.045×10^{-2}	1.457×10^{-2}	1.985×10^{-3}	3.848×10^{-3}	1.244×10^{-2}	1.842×10^{-2}	1.569×10^{-5}	2.186×10^{-5}	2.978×10^{-6}	5.771×10^{-6}	1.867×10^{-5}	2.763×10^{-5}
Cu	4.849×10^{-3}	6.757×10^{-3}	1.438×10^{-5}	2.787×10^{-5}	4.863×10^{-3}	6.784×10^{-3}						
Mn	4.481×10^{-5}	6.244×10^{-5}	3.101×10^{-5}	6.010×10^{-5}	7.582×10^{-5}	1.225×10^{-4}						
Ni	1.522×10^{-3}	2.121×10^{-3}	3.611×10^{-3}	6.999×10^{-3}	1.558×10^{-3}	2.191×10^{-3}	5.175×10^{-5}	7.211×10^{-5}	1.228×10^{-6}	2.380×10^{-6}	5.297×10^{-5}	7.449×10^{-5}
Co	1.719×10^{-5}	5.729×10^{-5}	1.170×10^{-4}	2.269×10^{-4}	1.342×10^{-4}	2.841×10^{-4}						
Al	3.616×10^{-1}	5.039×10^{-1}	3.432×10^{-6}	6.652×10^{-6}	3.616×10^{-1}	5.039×10^{-1}						
Pb	4.731×10^{-3}	6.593×10^{-3}	7.484×10^{-6}	1.450×10^{-5}	4.739×10^{-3}	6.607×10^{-3}	5.630×10^{-8}	7.846×10^{-8}	2.295×10^{10}	4.447×10^{10}	5.653×10^{-8}	7.890×10^{-8}
Hg	2.489×10^{-5}	3.469×10^{-5}	1.688×10^{-3}	3.271×10^{-3}	1.712×10^{-3}	3.305×10^{-3}						

(a) Wet season in R1

As	7.786×10^{-2}	1.085×10^{-1}	9.012×10^{-4}	1.747×10^{-3}	7.876×10^{-2}	1.102×10^{-1}	3.504×10^{-5}	4.883×10^{-5}	1.663×10^{-7}	3.222×10^{-7}	3.520×10^{-5}	4.915×10^{-5}
Zn	2.347×10^{-4}	7.825×10^{-4}	2.398×10^{-6}	4.648×10^{-6}	2.371×10^{-4}	7.871×10^{-4}						
Cr	6.789×10^{-3}	9.461×10^{-3}	1.289×10^{-3}	2.498×10^{-3}	8.078×10^{-3}	1.196×10^{-2}	1.018×10^{-5}	1.419×10^{-5}	1.933×10^{-6}	3.746×10^{-6}	1.212×10^{-5}	1.794×10^{-5}
Cu	5.045×10^{-3}	7.031×10^{-3}	1.496×10^{-5}	2.900×10^{-5}	5.060×10^{-3}	7.060×10^{-3}						
Mn	1.194×10^{-4}	1.665×10^{-4}	8.269×10^{-5}	1.603×10^{-4}	2.022×10^{-4}	3.268×10^{-4}						
Ni	1.318×10^{-3}	1.837×10^{-3}	3.127×10^{-5}	6.061×10^{-5}	1.349×10^{-3}	1.897×10^{-3}	4.481×10^{-5}	6.245×10^{-5}	1.063×10^{-6}	2.061×10^{-6}	4.588×10^{-5}	6.451×10^{-5}
Al	7.391×10^{0}	1.030×10^{1}	7.015×10^{-5}	1.359×10^{-4}	7.391×10^{0}	1.030×10^{0}						
Pb	1.592×10^{-3}	2.219×10^{-3}	2.519×10^{-6}	4.883×10^{-6}	1.595×10^{-3}	2.224×10^{-3}	1.895×10^{-8}	2.641×10^{-8}	7.724×10^{-11}	1.497×10^{-10}	1.903×10^{-8}	2.656×10^{-8}

（b）Dry season in R2

As	1.650×10^{-1}	2.300×10^{-1}	1.911×10^{-3}	3.703×10^{-3}	1.670×10^{-1}	2.337×10^{-1}	7.428×10^{-5}	1.035×10^{-4}	3.525×10^{-7}	6.832×10^{-7}	7.463×10^{-5}	1.041×10^{-4}
Cd	1.028×10^{-2}	1.432×10^{-2}	9.755×10^{-4}	1.891×10^{-3}	1.125×10^{-2}	1.621×10^{-2}	3.135×10^{-5}	4.368×10^{-5}	9.267×10^{-9}	1.796×10^{-8}	3.136×10^{-5}	4.370×10^{-5}
Zn	5.252×10^{-4}	1.751×10^{-3}	5.366×10^{-6}	1.040×10^{-5}	5.306×10^{-4}	1.761×10^{-3}						

Cr	1.187×10^{-2}	1.654×10^{-2}	2.254×10^{-3}	4.368×10^{-3}	1.413×10^{-2}	2.091×10^{-2}	1.781×10^{-5}	2.482×10^{-5}	3.380×10^{-6}	6.551×10^{-6}	2.119×10^{-5}	3.137×10^{-5}
Cu	3.777×10^{-3}	5.264×10^{-3}	1.120×10^{-5}	2.171×10^{-5}	3.789×10^{-3}	5.285×10^{-3}						
Mn	9.792×10^{-5}	1.364×10^{-4}	6.776×10^{-5}	1.313×10^{-4}	1.657×10^{-4}	2.678×10^{-4}						
Ni	1.659×10^{-3}	2.312×10^{-3}	3.937×10^{-5}	7.630×10^{-5}	1.699×10^{-3}	2.388×10^{-3}	5.641×10^{-5}	7.861×10^{-5}	1.339×10^{-6}	2.594×10^{-6}	5.775×10^{-5}	8.121×10^{-5}
Co	1.835×10^{-5}	6.117×10^{-5}	1.245×10^{-4}	2.422×10^{-4}	1.433×10^{-4}	3.034×10^{-4}						
Al	9.294×10^{-1}	1.295×10^{0}	8.821×10^{-6}	1.710×10^{-5}	9.294×10^{-1}	1.295×10^{0}						
Pb	3.802×10^{-3}	5.298×10^{-3}	6.014×10^{-6}	1.166×10^{-5}	3.808×10^{-3}	5.310×10^{-3}	4.525×10^{-8}	6.305×10^{-8}	1.844×10^{-10}	3.574×10^{-10}	4.543×10^{-8}	6.341×10^{-8}
Hg	3.286×10^{-5}	4.579×10^{-5}	2.228×10^{-3}	4.318×10^{-3}	2.261×10^{-3}	4.363×10^{-3}						

（d）Wet season in R2

As	7.210×10^{-2}	1.005×10^{-1}	8.345×10^{-4}	1.617×10^{-3}	7.293×10^{-2}	1.021×10^{-1}	3.244×10^{-5}	4.521×10^{-5}	1.540×10^{-7}	2.984×10^{-7}	3.260×10^{-5}	4.551×10^{-5}
Zn	1.565×10^{-4}	5.216×10^{-4}	1.599×10^{-6}	3.099×10^{-6}	1.581×10^{-4}	5.247×10^{-4}						
Cr	8.018×10^{-3}	1.117×10^{-2}	1.522×10^{-3}	2.950×10^{-3}	9.540×10^{-3}	1.412×10^{-2}	1.203×10^{-5}	1.676×10^{-5}	2.283×10^{-6}	4.425×10^{-6}	1.431×10^{-5}	2.119×10^{-5}
Cu	5.152×10^{-3}	7.180×10^{-3}	1.528×10^{-5}	2.962×10^{-5}	5.168×10^{-3}	7.210×10^{-3}						
Mn	1.562×10^{-4}	2.177×10^{-4}	1.081×10^{-4}	2.095×10^{-4}	2.643×10^{-4}	4.272×10^{-4}						
Ni	1.494×10^{-3}	2.081×10^{-3}	3.544×10^{-5}	6.869×10^{-5}	1.529×10^{-3}	2.150×10^{-3}	5.078×10^{-5}	7.077×10^{-5}	1.205×10^{-6}	2.335×10^{-6}	5.199×10^{-5}	7.310×10^{-5}
Al	6.685×10^{0}	9.316×10^{0}	6.345×10^{-5}	1.230×10^{-4}	6.686×10^{0}	9.316×10^{0}						
Pb	6.240×10^{-4}	8.697×10^{-4}	9.872×10^{-7}	1.913×10^{-6}	6.251×10^{-4}	8.716×10^{-4}	7.427×10^{-9}	1.035×10^{-8}	3.027×10^{-11}	5.866×10^{-11}	7.457×10^{-11}	1.041×10^{-8}

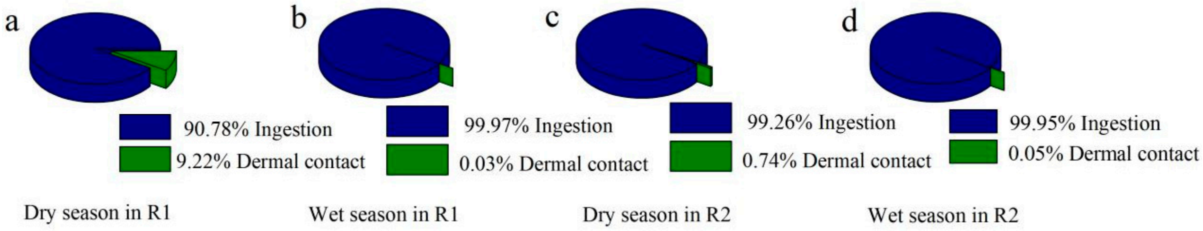

Figure S1: Contribution of various routes to HI. (a) Contribution of ingestion and dermal pathways at the R1 sampling site during the dry season; (b) Contribution of ingestion and dermal pathways at sampling point R1 in the wet season; (c) Contribution of ingestion and dermal pathways at sampling point R2 during the dry season; (d) Contribution of ingestion and dermal pathways at sampling point R2 during the wet season.

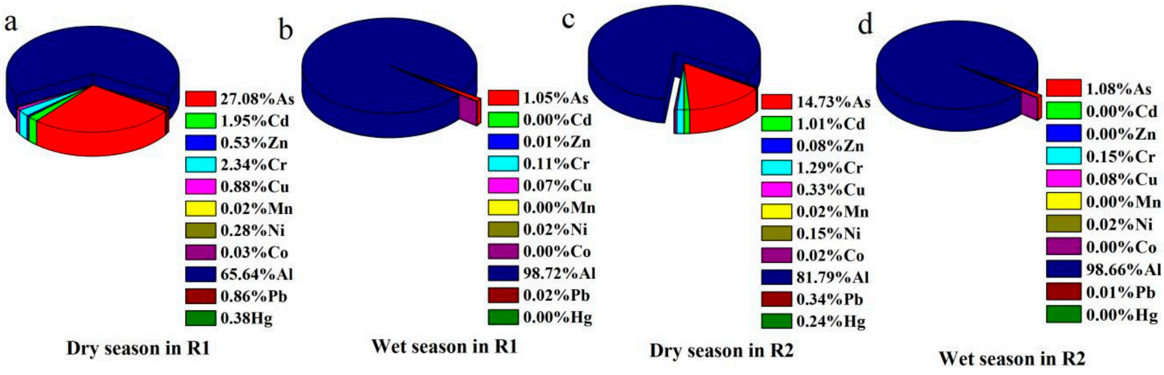

Figure S2: Contribution of different metals to HI in sample station. (a) Contribution of different metals to HI at the R1 sampling site during the dry season; (b) Contribution of different metals to HI at sampling point R1 in the wet season; (c) Contribution of different metals to HI at sampling point R2 during the dry season; (d) Contribution of different metals to HI at sampling point R2 during the wet season.
